# Bis(2-amino­pyrimidine-κ*N* ^1^)aqua­(nitrato-κ*O*)(nitrato-κ^2^ *O*,*O*′)zinc(II)

**DOI:** 10.1107/S1600536810036731

**Published:** 2010-09-18

**Authors:** Shan Gao, Seik Weng Ng

**Affiliations:** a College of Chemistry and Materials Science, Heilongjiang University, Harbin 150080, People’s Republic of China; b Department of Chemistry, University of Malaya, 50603 Kuala Lumpur, Malaysia

## Abstract

The water-coordinated Zn atom in the title monoaqua zinc nitrate adduct of 2-amino­pyrimidine, [Zn(NO_3_)_2_(C_4_H_5_N_3_)_2_(H_2_O)], is bonded to a monodentate nitrate ion and is chelated by the other nitrate ion. The heterocyclic ligands coordinate through ring *N*-donor sites. The coordination geometry about the Zn(II) atom is a distorted octa­hedron. Intra­molecular N—H⋯O hydrogen bonds occur. In the crystal, adjacent adduct mol­ecules are linked by O—H⋯O, O—H⋯N and N—H⋯O hydrogen bonds into a layer motif parallel to (001).

## Related literature

The aqua­zinc nitrate adduct is isotypic with its Co and Ni analogs, see: Pike *et al.* (2006 ▶). The copper nitrate adduct is anhydrous, see: Albada *et al.* (2002 ▶).
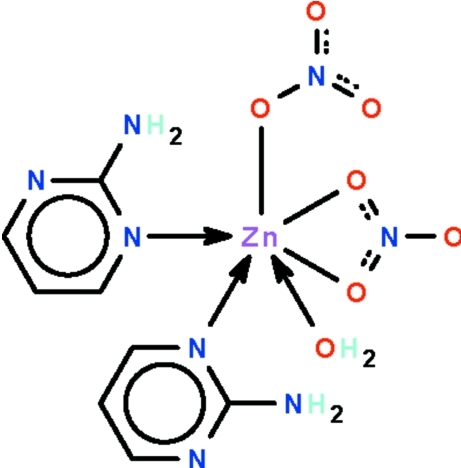


## Experimental

### 

#### Crystal data

[Zn(NO_3_)_2_(C_4_H_5_N_3_)_2_(H_2_O)]
*M* *_r_* = 397.63
Monoclinic, 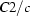

*a* = 13.2742 (4) Å
*b* = 8.0142 (2) Å
*c* = 28.6204 (7) Å
β = 101.335 (1)°
*V* = 2985.31 (14) Å^3^
*Z* = 8
Mo *K*α radiation
μ = 1.70 mm^−1^
*T* = 293 K
0.22 × 0.18 × 0.12 mm

#### Data collection

Rigaku R-AXIS RAPID diffractometer
Absorption correction: multi-scan (*ABSCOR*; Higashi, 1995 ▶) *T*_min_ = 0.706, *T*_max_ = 0.822
14113 measured reflections
3401 independent reflections
3006 reflections with *I* > 2σ(*I*)
*R*_int_ = 0.039

#### Refinement

*R*[*F* ^2^ > 2σ(*F* ^2^)] = 0.030
*wR*(*F* ^2^) = 0.081
*S* = 1.04
3401 reflections
241 parameters
6 restraints
H atoms treated by a mixture of independent and constrained refinement
Δρ_max_ = 0.40 e Å^−3^
Δρ_min_ = −0.43 e Å^−3^

### 

Data collection: *RAPID-AUTO* (Rigaku, 1998 ▶); cell refinement: *RAPID-AUTO*; data reduction: *CrystalStructure* (Rigaku/MSC and Rigaku, 2002 ▶); program(s) used to solve structure: *SHELXS97* (Sheldrick, 2008 ▶); program(s) used to refine structure: *SHELXL97* (Sheldrick, 2008 ▶); molecular graphics: *X-SEED* (Barbour, 2001 ▶); software used to prepare material for publication: *publCIF* (Westrip, 2010 ▶).

## Supplementary Material

Crystal structure: contains datablocks global, I. DOI: 10.1107/S1600536810036731/jh2204sup1.cif

Structure factors: contains datablocks I. DOI: 10.1107/S1600536810036731/jh2204Isup2.hkl

Additional supplementary materials:  crystallographic information; 3D view; checkCIF report

## Figures and Tables

**Table 1:** Hydrogen-bond geometry (Å, °)

*D*—H⋯*A*	*D*—H	H⋯*A*	*D*⋯*A*	*D*—H⋯*A*
O1w—H11⋯O2^i^	0.83 (1)	1.99 (2)	2.776 (2)	158 (3)
O1w—H12⋯N2^ii^	0.84 (1)	1.94 (1)	2.754 (2)	165 (3)
N3—H31⋯O1	0.87 (1)	2.23 (2)	2.989 (3)	146 (2)
N3—H32⋯O5^iii^	0.86 (1)	2.34 (2)	3.133 (3)	152 (3)
N6—H61⋯O1	0.87 (1)	2.37 (3)	3.010 (2)	131 (3)
N6—H61⋯O5	0.87 (1)	2.43 (2)	3.122 (3)	137 (3)
N6—H62⋯O1^iv^	0.87 (1)	2.41 (2)	3.192 (2)	150 (3)
N6—H62⋯O3^iv^	0.87 (1)	2.45 (2)	3.265 (3)	156 (3)

Symmetry codes: (i) 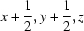; (ii) 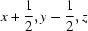; (iii) 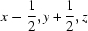; (iv) 
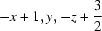
.

**Fig. 1.**
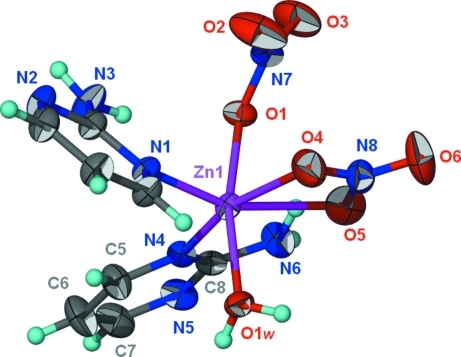
Thermal ellipsoid plot (Barbour, 2001) of Zn(H2O)(NO3)2(C4H5N3)2 at the 50% probability level; hydrogen atoms are drawn as spheres of arbitrary radius.

**Fig. 2.**
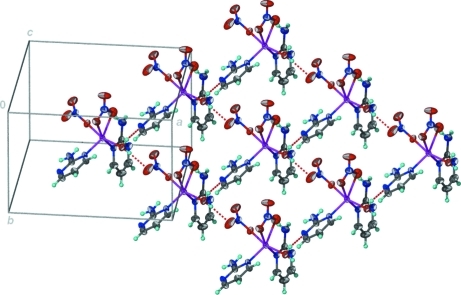
Hydrogen-bonded layer structure.

## supplementary crystallographic information

### Comment

The cobalt, nickel and copper adducts of 2-aminopyrimidine have been reported; the first two are monoaqua complexes (Pike *et al.*, 2006) whereas the copper complex is anhydrous (Albada *et al.*, 2002). In the aqua complexes, one nitrate is monodentate and the other is chelating; the heterocyclic ligand coordinates through a ring donor site. The present zinc analog (Scheme I, Fig. 1) is isostructural to the cobalt and nickel adducts, whose structures have been described in detail. Adjacent molecules are linked by O–H···O and N–H···O hydrogen bonds into a layer motif (Fig. 2).

### Experimental

Zinc nitrate (1 mmol) and 2-aminopyrimidine (1 mmol) were dissolved in a small volume of water to give a colorless solution. Colorless prismatic crystals separated from the solution after a few days.

### Refinement

Carbon-bound H-atoms were placed in calculated positions (C—H 0.93 Å) and were included in the refinement in the riding model approximation, with *U*(H) set to 1.2*U*(C).

The amino and water H-atoms were located in a difference Fourier map, and were refined with a distance restraint of N–H 0.88±0.01 and O–H 0.84±0.01 Å; their temperature factors were freely refined.

### Crystal data

**Table:**

[Zn(NO_3_)_2_(C_4_H_5_N_3_)_2_(H_2_O)]	*F*(000) = 1616
*M**_r_* = 397.63	*D*_x_ = 1.769 Mg m^−^^3^
Monoclinic, *C*2/*c*	Mo *K*α radiation, λ = 0.71073 Å
Hall symbol: -C 2yc	Cell parameters from 11713 reflections
*a* = 13.2742 (4) Å	θ = 3.0–27.4°
*b* = 8.0142 (2) Å	µ = 1.70 mm^−^^1^
*c* = 28.6204 (7) Å	*T* = 293 K
β = 101.335 (1)°	Prism, colorless
*V* = 2985.31 (14) Å^3^	0.22 × 0.18 × 0.12 mm
*Z* = 8	

### Data collection

**Table:**

Rigaku R-AXIS RAPID diffractometer	3401 independent reflections
Radiation source: fine-focus sealed tube	3006 reflections with *I* > 2σ(*I*)
graphite	*R*_int_ = 0.039
Detector resolution: 10.000 pixels mm^-1^	θ_max_ = 27.4°, θ_min_ = 3.0°
ω scans	*h* = −17→17
Absorption correction: multi-scan (*ABSCOR*; Higashi, 1995)	*k* = −10→10
*T*_min_ = 0.706, *T*_max_ = 0.822	*l* = −34→37
14113 measured reflections	

### Refinement

**Table:**

Refinement on *F*^2^	Primary atom site location: structure-invariant direct methods
Least-squares matrix: full	Secondary atom site location: difference Fourier map
*R*[*F*^2^ > 2σ(*F*^2^)] = 0.030	Hydrogen site location: inferred from neighbouring sites
*wR*(*F*^2^) = 0.081	H atoms treated by a mixture of independent and constrained refinement
*S* = 1.04	*w* = 1/[σ^2^(*F*_o_^2^) + (0.0479*P*)^2^ + 2.0445*P*] where *P* = (*F*_o_^2^ + 2*F*_c_^2^)/3
3401 reflections	(Δ/σ)_max_ = 0.001
241 parameters	Δρ_max_ = 0.40 e Å^−^^3^
6 restraints	Δρ_min_ = −0.42 e Å^−^^3^

### Fractional atomic coordinates and isotropic or equivalent isotropic displacement parameters (Å^2^)

**Table:**

	*x*	*y*	*z*	*U*_iso_*/*U*_eq_	
Zn1	0.498530 (14)	0.66515 (3)	0.616791 (7)	0.02883 (9)	
O1	0.39847 (10)	0.55960 (19)	0.66269 (5)	0.0379 (3)	
O2	0.28031 (17)	0.4531 (3)	0.60891 (6)	0.0822 (7)	
O3	0.30770 (17)	0.3540 (3)	0.67905 (8)	0.0742 (6)	
O4	0.46872 (15)	0.4598 (2)	0.55893 (9)	0.0764 (7)	
O5	0.56026 (15)	0.4082 (3)	0.62668 (7)	0.0614 (5)	
O6	0.5176 (2)	0.2078 (2)	0.57552 (7)	0.0745 (6)	
O1W	0.62147 (10)	0.7246 (2)	0.58475 (5)	0.0377 (3)	
N1	0.39392 (11)	0.8157 (2)	0.57361 (5)	0.0301 (3)	
N2	0.24514 (12)	0.9882 (2)	0.55462 (6)	0.0398 (4)	
N3	0.29718 (15)	0.8839 (3)	0.63018 (7)	0.0492 (5)	
N4	0.55915 (12)	0.8053 (2)	0.67664 (5)	0.0306 (3)	
N5	0.64072 (15)	0.8410 (2)	0.75816 (6)	0.0437 (4)	
N6	0.60734 (16)	0.5800 (2)	0.72650 (7)	0.0460 (4)	
N7	0.32868 (12)	0.4509 (2)	0.64962 (6)	0.0371 (4)	
N8	0.51533 (15)	0.3535 (2)	0.58614 (8)	0.0466 (4)	
C1	0.40689 (15)	0.8333 (3)	0.52831 (7)	0.0351 (4)	
H1	0.4624	0.7805	0.5192	0.042*	
C2	0.34156 (16)	0.9258 (3)	0.49524 (7)	0.0415 (5)	
H2	0.3512	0.9366	0.4641	0.050*	
C3	0.26084 (16)	1.0021 (3)	0.51044 (7)	0.0418 (5)	
H3	0.2154	1.0662	0.4888	0.050*	
C4	0.31217 (13)	0.8944 (3)	0.58546 (7)	0.0329 (4)	
C5	0.56075 (18)	0.9717 (3)	0.67104 (8)	0.0447 (5)	
H5	0.5335	1.0169	0.6413	0.054*	
C6	0.6010 (2)	1.0766 (3)	0.70754 (10)	0.0586 (6)	
H6	0.6024	1.1916	0.7033	0.070*	
C7	0.63959 (19)	1.0039 (3)	0.75104 (8)	0.0534 (6)	
H7	0.6661	1.0729	0.7766	0.064*	
C8	0.60164 (13)	0.7461 (3)	0.72035 (6)	0.0317 (4)	
H11	0.6657 (17)	0.789 (3)	0.5994 (9)	0.061 (8)*	
H12	0.653 (2)	0.640 (2)	0.5780 (11)	0.067 (9)*	
H31	0.3355 (18)	0.817 (3)	0.6501 (8)	0.053 (8)*	
H32	0.2393 (12)	0.920 (3)	0.6356 (10)	0.057 (8)*	
H61	0.567 (2)	0.515 (3)	0.7066 (9)	0.074 (9)*	
H62	0.624 (2)	0.548 (4)	0.7560 (5)	0.071 (9)*	

### Atomic displacement parameters (Å^2^)

**Table:**

	*U*^11^	*U*^22^	*U*^33^	*U*^12^	*U*^13^	*U*^23^
Zn1	0.02802 (13)	0.03286 (14)	0.02437 (13)	0.00355 (8)	0.00208 (8)	−0.00019 (8)
O1	0.0338 (7)	0.0452 (8)	0.0339 (7)	−0.0098 (6)	0.0044 (5)	−0.0037 (6)
O2	0.0895 (14)	0.1073 (18)	0.0386 (9)	−0.0538 (13)	−0.0145 (9)	0.0059 (10)
O3	0.0787 (14)	0.0756 (14)	0.0671 (13)	−0.0275 (10)	0.0110 (11)	0.0237 (10)
O4	0.0554 (10)	0.0468 (10)	0.1130 (18)	0.0005 (8)	−0.0180 (11)	0.0186 (11)
O5	0.0691 (11)	0.0716 (12)	0.0484 (10)	−0.0027 (9)	0.0233 (8)	−0.0121 (9)
O6	0.1327 (19)	0.0331 (9)	0.0651 (12)	0.0095 (10)	0.0380 (12)	−0.0043 (9)
O1W	0.0294 (7)	0.0403 (8)	0.0446 (8)	−0.0014 (6)	0.0102 (6)	−0.0080 (7)
N1	0.0258 (7)	0.0392 (9)	0.0245 (7)	0.0043 (6)	0.0032 (6)	0.0006 (6)
N2	0.0365 (8)	0.0476 (10)	0.0336 (8)	0.0150 (7)	0.0028 (7)	0.0005 (7)
N3	0.0460 (10)	0.0729 (13)	0.0310 (9)	0.0265 (10)	0.0133 (8)	0.0088 (9)
N4	0.0319 (7)	0.0340 (8)	0.0257 (7)	−0.0031 (6)	0.0051 (6)	0.0003 (6)
N5	0.0488 (10)	0.0522 (11)	0.0279 (8)	−0.0134 (8)	0.0022 (7)	−0.0063 (7)
N6	0.0556 (11)	0.0426 (10)	0.0327 (9)	−0.0036 (8)	−0.0089 (8)	0.0065 (8)
N7	0.0328 (8)	0.0439 (9)	0.0345 (8)	−0.0068 (7)	0.0066 (6)	0.0001 (7)
N8	0.0512 (11)	0.0366 (10)	0.0561 (12)	0.0037 (8)	0.0210 (9)	−0.0023 (8)
C1	0.0344 (9)	0.0444 (11)	0.0272 (9)	0.0067 (8)	0.0075 (7)	0.0022 (7)
C2	0.0451 (11)	0.0533 (13)	0.0256 (9)	0.0084 (9)	0.0056 (8)	0.0066 (9)
C3	0.0412 (10)	0.0475 (12)	0.0330 (10)	0.0103 (9)	−0.0018 (8)	0.0042 (9)
C4	0.0296 (9)	0.0401 (10)	0.0281 (9)	0.0053 (7)	0.0030 (7)	−0.0011 (7)
C5	0.0589 (13)	0.0350 (11)	0.0386 (11)	−0.0020 (9)	0.0055 (9)	0.0039 (9)
C6	0.0835 (18)	0.0343 (12)	0.0553 (14)	−0.0100 (11)	0.0072 (13)	−0.0061 (10)
C7	0.0634 (14)	0.0536 (14)	0.0417 (12)	−0.0178 (12)	0.0068 (10)	−0.0162 (11)
C8	0.0287 (8)	0.0404 (11)	0.0252 (8)	−0.0058 (7)	0.0035 (7)	−0.0001 (7)

### Geometric parameters (Å, °)

**Table:**

Zn1—N1	2.0583 (15)	N3—H31	0.869 (10)
Zn1—N4	2.0740 (16)	N3—H32	0.864 (10)
Zn1—O1W	2.0782 (14)	N4—C5	1.343 (3)
Zn1—O5	2.214 (2)	N4—C8	1.353 (2)
Zn1—O1	2.2117 (14)	N5—C7	1.320 (3)
Zn1—O4	2.313 (2)	N5—C8	1.341 (2)
O1—N7	1.273 (2)	N6—C8	1.342 (3)
O2—N7	1.215 (2)	N6—H61	0.871 (10)
O3—N7	1.218 (3)	N6—H62	0.869 (10)
O4—N8	1.235 (3)	C1—C2	1.369 (3)
O5—N8	1.274 (3)	C1—H1	0.9300
O6—N8	1.209 (2)	C2—C3	1.376 (3)
O1W—H11	0.831 (10)	C2—H2	0.9300
O1W—H12	0.840 (10)	C3—H3	0.9300
N1—C1	1.348 (2)	C5—C6	1.366 (3)
N1—C4	1.355 (2)	C5—H5	0.9300
N2—C3	1.326 (3)	C6—C7	1.379 (4)
N2—C4	1.351 (2)	C6—H6	0.9300
N3—C4	1.336 (3)	C7—H7	0.9300
			
N1—Zn1—N4	106.55 (6)	C8—N6—H62	115 (2)
N1—Zn1—O1W	95.52 (6)	H61—N6—H62	118 (3)
N4—Zn1—O1W	91.68 (6)	O3—N7—O2	121.54 (19)
N1—Zn1—O5	144.20 (7)	O3—N7—O1	119.02 (18)
N4—Zn1—O5	108.93 (7)	O2—N7—O1	119.28 (18)
O1W—Zn1—O5	88.09 (6)	O6—N8—O4	122.8 (2)
N1—Zn1—O1	99.66 (6)	O6—N8—O5	122.1 (2)
N4—Zn1—O1	84.11 (6)	O4—N8—O5	115.1 (2)
O1W—Zn1—O1	164.82 (6)	N1—C1—C2	122.55 (18)
O5—Zn1—O1	79.56 (6)	N1—C1—H1	118.7
N1—Zn1—O4	89.25 (6)	C2—C1—H1	118.7
N4—Zn1—O4	163.88 (6)	C1—C2—C3	116.67 (19)
O1W—Zn1—O4	83.45 (7)	C1—C2—H2	121.7
O5—Zn1—O4	55.71 (7)	C3—C2—H2	121.7
O1—Zn1—O4	96.60 (7)	N2—C3—C2	122.81 (18)
N7—O1—Zn1	125.05 (12)	N2—C3—H3	118.6
N8—O4—Zn1	92.60 (16)	C2—C3—H3	118.6
N8—O5—Zn1	96.21 (14)	N3—C4—N2	117.25 (17)
Zn1—O1W—H11	117 (2)	N3—C4—N1	119.20 (17)
Zn1—O1W—H12	113 (2)	N2—C4—N1	123.53 (17)
H11—O1W—H12	106 (3)	N4—C5—C6	122.2 (2)
C1—N1—C4	116.87 (16)	N4—C5—H5	118.9
C1—N1—Zn1	116.11 (12)	C6—C5—H5	118.9
C4—N1—Zn1	126.99 (13)	C5—C6—C7	116.8 (2)
C3—N2—C4	117.57 (17)	C5—C6—H6	121.6
C4—N3—H31	119.3 (18)	C7—C6—H6	121.6
C4—N3—H32	117.1 (19)	N5—C7—C6	123.2 (2)
H31—N3—H32	121 (3)	N5—C7—H7	118.4
C5—N4—C8	116.43 (17)	C6—C7—H7	118.4
C5—N4—Zn1	116.84 (13)	N6—C8—N5	117.00 (17)
C8—N4—Zn1	126.66 (13)	N6—C8—N4	118.12 (17)
C7—N5—C8	116.47 (19)	N5—C8—N4	124.86 (19)
C8—N6—H61	120 (2)		
			
N1—Zn1—O1—N7	72.86 (16)	O5—Zn1—N4—C8	−19.60 (17)
N4—Zn1—O1—N7	178.70 (16)	O1—Zn1—N4—C8	57.21 (15)
O1W—Zn1—O1—N7	−106.8 (2)	O4—Zn1—N4—C8	−36.2 (3)
O5—Zn1—O1—N7	−70.77 (15)	Zn1—O1—N7—O3	150.33 (18)
O4—Zn1—O1—N7	−17.50 (16)	Zn1—O1—N7—O2	−34.3 (3)
N1—Zn1—O4—N8	−168.46 (15)	Zn1—O4—N8—O6	173.3 (2)
N4—Zn1—O4—N8	22.8 (4)	Zn1—O4—N8—O5	−5.9 (2)
O1W—Zn1—O4—N8	95.89 (15)	Zn1—O5—N8—O6	−173.0 (2)
O5—Zn1—O4—N8	3.73 (13)	Zn1—O5—N8—O4	6.2 (2)
O1—Zn1—O4—N8	−68.82 (15)	C4—N1—C1—C2	0.0 (3)
N1—Zn1—O5—N8	9.79 (19)	Zn1—N1—C1—C2	178.16 (17)
N4—Zn1—O5—N8	−178.14 (12)	N1—C1—C2—C3	0.3 (3)
O1W—Zn1—O5—N8	−87.01 (13)	C4—N2—C3—C2	0.0 (3)
O1—Zn1—O5—N8	101.86 (13)	C1—C2—C3—N2	−0.3 (4)
O4—Zn1—O5—N8	−3.63 (13)	C3—N2—C4—N3	178.6 (2)
N4—Zn1—N1—C1	126.33 (14)	C3—N2—C4—N1	0.4 (3)
O1W—Zn1—N1—C1	32.92 (15)	C1—N1—C4—N3	−178.6 (2)
O5—Zn1—N1—C1	−61.49 (19)	Zn1—N1—C4—N3	3.6 (3)
O1—Zn1—N1—C1	−147.00 (14)	C1—N1—C4—N2	−0.4 (3)
O4—Zn1—N1—C1	−50.43 (15)	Zn1—N1—C4—N2	−178.24 (15)
N4—Zn1—N1—C4	−55.78 (17)	C8—N4—C5—C6	−1.3 (3)
O1W—Zn1—N1—C4	−149.19 (16)	Zn1—N4—C5—C6	−178.4 (2)
O5—Zn1—N1—C4	116.40 (17)	N4—C5—C6—C7	−0.6 (4)
O1—Zn1—N1—C4	30.90 (17)	C8—N5—C7—C6	−0.1 (4)
O4—Zn1—N1—C4	127.46 (17)	C5—C6—C7—N5	1.3 (4)
N1—Zn1—N4—C5	−27.69 (17)	C7—N5—C8—N6	176.5 (2)
O1W—Zn1—N4—C5	68.58 (16)	C7—N5—C8—N4	−2.1 (3)
O5—Zn1—N4—C5	157.14 (15)	C5—N4—C8—N6	−175.85 (19)
O1—Zn1—N4—C5	−126.05 (16)	Zn1—N4—C8—N6	0.9 (3)
O4—Zn1—N4—C5	140.6 (3)	C5—N4—C8—N5	2.8 (3)
N1—Zn1—N4—C8	155.57 (14)	Zn1—N4—C8—N5	179.52 (15)
O1W—Zn1—N4—C8	−108.16 (15)		

### Hydrogen-bond geometry (Å, °)

**Table:**

*D*—H···*A*	*D*—H	H···*A*	*D*···*A*	*D*—H···*A*
O1w—H11···O2^i^	0.83 (1)	1.99 (2)	2.776 (2)	158 (3)
O1w—H12···N2^ii^	0.84 (1)	1.94 (1)	2.754 (2)	165 (3)
N3—H31···O1	0.87 (1)	2.23 (2)	2.989 (3)	146 (2)
N3—H32···O5^iii^	0.86 (1)	2.34 (2)	3.133 (3)	152 (3)
N6—H61···O1	0.87 (1)	2.37 (3)	3.010 (2)	131 (3)
N6—H61···O5	0.87 (1)	2.43 (2)	3.122 (3)	137 (3)
N6—H62···O1^iv^	0.87 (1)	2.41 (2)	3.192 (2)	150 (3)
N6—H62···O3^iv^	0.87 (1)	2.45 (2)	3.265 (3)	156 (3)

Symmetry codes: (i) *x*+1/2, *y*+1/2, *z*; (ii) *x*+1/2, *y*−1/2, *z*; (iii) *x*−1/2, *y*+1/2, *z*; (iv) −*x*+1, *y*, −*z*+3/2.
